# Bridging Mammography and Lung Cancer Screening: Eligibility, Uptake and Potential Impact

**DOI:** 10.1002/cam4.71528

**Published:** 2026-01-20

**Authors:** Ali Ajrouch, Yara Khalifeh, Amir F. Beirat, Dana Alhaffar, Ahmad Karkash, Razan Aljaras, Adel Hajj Ali, Nicholas Pettit, Deanna R. Willis, Victoria L. Champion, Lisa Carter‐Bawa, Amrou Awaysheh, Kolawole S. Okuyemi

**Affiliations:** ^1^ Department of Family Medicine Indiana University School of Medicine Indianapolis Indiana USA; ^2^ Department of Internal Medicine Indiana University School of Medicine Indianapolis Indiana USA; ^3^ Department of Emergency Medicine Indiana University School of Medicine Indianapolis Indiana USA; ^4^ School of Nursing Indiana University Indianapolis Indiana USA; ^5^ Cancer Prevention Precision Control Institute Center for Discovery & Innovation at Hackensack Meridian Health Nutley New Jersey USA; ^6^ Cancer Prevention & Control Program Georgetown Lombardi Comprehensive Cancer Center Consortium Washington DC USA; ^7^ Kelley School of Business Indiana University Indianapolis Indiana USA

**Keywords:** health disparities, lung cancer, lung cancer screening, mammography, market viability assessment, women’s health

## Abstract

**Introduction:**

Lung cancer (LC) is the top cancer killer in women, yet lung cancer screening (LCS) uptake is substantially lower than mammography. Leveraging the reach of mammography programs may improve LCS uptake, but the potential gain in LC detection from this approach is unknown. This study aimed to determine the proportion of women with LC eligible for both screenings, potential LC detection via integrated screening, and factors influencing each screening uptake among those dually eligible.

**Methods:**

This retrospective cross‐sectional study included 345 women newly diagnosed with LC presenting at a Midwestern Comprehensive Cancer Center (2019–2020). Pre‐diagnosis LCS‐eligibility was determined per 2013 and 2021 USPSTF criteria, LCS‐uptake per 2013 criteria, and mammography‐eligibility per 2016 criteria. We assessed sociodemographic variables associated with screening uptake among dually eligible women.

**Results:**

Among 345 women (mean [SD] age 64.8 [11.35] years), 73.3% were eligible for mammography, while 43.5% were eligible for LCS (2013), increasing to 49.3% (2021). Mammography uptake (41.5%) substantially exceeded LCS uptake (13.9%). Overall, 45.2% were eligible for both screenings, representing 92.4% (157/170) of all LCS‐eligible (2021) cases. Notably, 20.3% were LCS‐eligible (2021) and received mammography, that is, 41.2% (70/170) of LCS‐eligible cases. Among dually eligible women, rural residency correlated with lower LCS uptake (odds ratio [OR], 0.42; 95% CI = 0.19–0.94; *p* = 0.031), whereas receiving mammography correlated with higher LCS uptake (OR, 2.67; 95% CI = 1.21–5.87; *p* = 0.013).

**Conclusion:**

A substantial proportion of women with LC who are LCS‐eligible underwent mammography, representing a missed opportunity for earlier LC detection. Integrating these screenings could enhance LC detection, especially for rural residents who experience disparities in LCS but not mammography uptake.

## 1 Introduction

Lung cancer (LC) is a critical but underrecognized women's health issue [1]. As the leading cause of cancer‐related deaths in the United States (US), it accounts for more mortalities than breast and ovarian cancer combined [2, 3]. This high mortality rate is largely preventable by early detection [1, 4]. Yet only 18% of eligible women undergo lung cancer screening (LCS) with low‐dose computed tomography (CT), compared to 76% for mammography [3, 5]. This disparity is starkest in underserved populations such as racial and gender minorities [6]. These groups experience greater LC mortality due to higher LC incidence from disproportionate exposure to cigarette smoke and environmental pollutants and greater barriers to LCS such as limited awareness of LCS, smoking‐related stigma, healthcare system mistrust, and a lack of physician recommendation [7, 8].

Many underserved groups who underutilize LCS commonly engage in mammography, suggesting an opportunity to leverage existing health‐seeking behavior and mammography programs' reach to improve LC early detection and reduce disparities. For example, Black Americans face higher LC mortality and lower LCS participation than White Americans, but higher mammography participation [3, 9, 10]. Similarly, lesbian and bisexual women are twice as likely as heterosexual women to develop LC due to higher smoking prevalence and intensity [11]. Although they undergo mammography at similar rates, they are three times less likely to undergo LCS [3, 12]. Additional evidence supports integrating these screening programs. The American Cancer Society (ACS) mammography program directors and the ACS National Lung Cancer Roundtable recognize the benefits of bridging these screenings but cite resource concerns [13, 14]. Additionally, LCS‐eligible mammography participants reported an interest in receiving LCS when informed about it [15, 16]. This receptiveness aligns with research showing that mammography participants are more likely to engage in additional cancer‐preventative care [17].

However, whether bridging LCS programs with mammography will increase LC detection is unknown. Previous research measured the potential of bridging these screenings by determining LCS eligibility and uptake rates in mammography participants [15, 18, 19]. However, these rates do not provide the real‐world effectiveness of the intervention‐the number of detectable LC cases nor the percentage they constitute out of all LC cases among women [20]. Addressing this gap is essential for determining whether bridging LCS and mammography would produce a sufficiently large benefit to justify the resource allocation. Additionally, the barriers and facilitators to engaging in LCS among eligible mammography participants diagnosed with LC are poorly understood. These measures are part of a “Market Viability Analysis,” a critical component of implementation science that quantifies the potential real‐world impact of an intervention before implementation to assess its feasibility and merit and identify the adopters of the innovation [21]. This approach is particularly valuable when evaluating resource‐intensive health interventions like cancer screening programs.

We conducted a retrospective cross‐sectional study of women newly diagnosed with LC to inform this potential practice innovation. We aimed to (1) determine the rates of singular and dual LCS and mammography eligibility and uptake among women with LC before their LC diagnosis; (2) quantify the percentage of LC cases that could be detected by promoting LCS among LCS‐eligible mammography participants who had not undergone LCS; and (3) identify the factors influencing screening uptake in patients dually eligible for both screenings. Understanding these dynamics is crucial for guiding intervention design, aiming to reduce LC mortality and disparities by leveraging a widely accepted and accessible cancer screening platform.

## 2 Methods

### 2.1 Study Design and Setting

We conducted a retrospective cross‐sectional study, manually collecting Electronic Health Record (EHR) data from all women newly diagnosed with primary LC and seen at a Midwestern Comprehensive Cancer Center between January 2019 and December 2020. This study was approved by the University Institutional Review Board (IRB #2001716264) and issued a waiver of informed consent. This paper follows the Strengthening the Reporting of Observational Studies in Epidemiology guidelines for reporting observational studies.

### 2.2 Population

**FIGURE 1:**
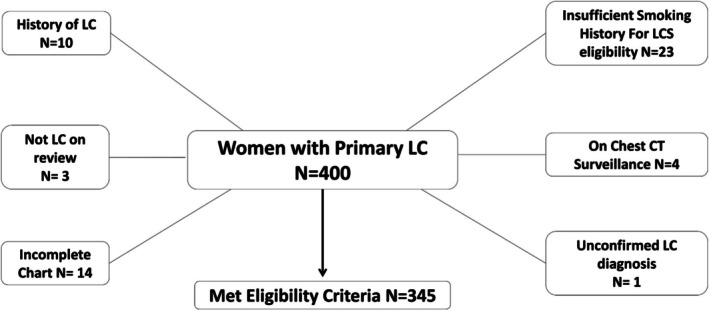
Diagram of patient selection. Out of 400 women initially identified with primary LC during the study period, we excluded a total of 55 patients who met one or more of the following criteria: Those with a prior LC diagnosis (*n* = 10), those without confirmed LC upon chart review (*n* = 3), those with substantially incomplete medical records (*n* = 14), those with insufficient smoking history to determine LCS eligibility (*n* = 23), patients under active chest CT surveillance (*n* = 4), and those without a biopsy‐confirmed LC diagnosis (*n* = 1). Patients were classified under the first ineligibility criteria they met. The final eligible cohort included 345 women. LC, lung cancer; LCS, lung cancer screening.

Using the center's tumor registry, we identified women aged ≥ 18 years with a newly diagnosed primary LC during the study period. We excluded patients with a prior LC diagnosis (as this would confound screening behavior, screening histories and the new LC diagnosis date), those under active chest CT surveillance (as they are already monitored and do not represent a true screening population), cases without biopsy‐confirmed LC diagnosis (to avoid misclassification of patients who might have had a different diagnosis or a false‐positive finding), patients with insufficient smoking history data to ascertain LCS eligibility when age‐appropriate and those whose records are missing over 60% of the information collected for this study beyond smoking history (Figure 1).

### 2.3 Data Collection

Trained abstractors manually reviewed the Cerner electronic health record (EHR) system and available outside records through the Indiana Network for Patient Care (INPC) Health Information Exchange [22]. It connects 80 clinical institutions covering the whole state and includes more than 25 million patients beginning from the 1990s. We defined all variables at the start of the study to ensure consistency. We implemented a double‐review process for quality assurance, where each abstractor reviewed another's data extraction for the first 10 charts. The team discussed and resolved discrepancies as they came up and maintained a decision log for consistent handling of edge cases. An independent third reviewer conducted checks on 80% of the first 50 entries, then randomly throughout the project for quality and completeness. We securely stored data in Research Electronic Data Capture (REDCap) [23].

- *Demographic and Clinical Variables*: We collected age, race, ethnicity, marital status, employment status, insurance status and type, and LC characteristics (stage, histology, and diagnosis date) at diagnosis or the closest available time point from “facesheets” and clinical notes/reports.
- *Smoking History*: We collected smoking amount (packs per day), duration (years), pack‐years, current smoking status, and quit date when applicable. When discrepancies were noted across different clinical notes (e.g., varying pack‐year totals), abstractors followed a standardized protocol: explicitly stated pack‐year calculations were prioritized. If pack‐years were not explicitly stated, they were calculated using the most consistently reported smoking intensity (packs per day) and duration (years smoked). The smoking status (current, former, or never) and any documented quit date were extracted from this same primary reference time point. For former smokers, “years since quitting” was calculated as the time between the documented quit date and the date of LC diagnosis. When structured data were incomplete, we used Cerner's chart search function to identify smoking information in clinical notes using standardized search terms (e.g., cigarette, quit, smoke, tobacco, pack‐years). The term “PPD” (pack per day) by itself was associated with tuberculosis as it is also the name of the tuberculosis skin test. Hence, it was qualified by being associated with terms such as “smoking, smokes, tobacco, or cigarettes.” When unspecific terms to determine lifetime pack years, such as “less than 10 pack years,” were used, we categorized patients under “simplified pack year” (Never Smoked; < 20 pack‐years; 20–30 pack‐years; ≥ 30 pack‐years; Unknown).
- *Screening Eligibility and Utilization*: LCS eligibility was defined based on the 2013 and 2021 US Preventive Services Task Force (USPSTF) guidelines, respectively and determined based on age and smoking history [24, 25]. The 2013 guidelines were used to reflect eligibility criteria at the time of patient diagnosis and screening (2019–2020), while the 2021 guidelines were applied to the cohort to understand the potential impact of the updated recommendations on eligibility numbers. *The 2013 USPSTF LCS guidelines recommended annual screening for adults aged 55–80 years who have a 30 pack‐year smoking history and currently smoke or have quit within the past 15 years*. *The 2021 USPSTF LCS guidelines expanded these criteria, recommending annual screening for adults aged 50–80 years who have a 20 pack‐year smoking history and currently smoke or have quit within the past 15 years*. Even with incomplete information, if the patient failed to meet one of the LCS eligibility criteria, such as age, pack years, or quit duration, they were classified as ineligible and included in the analysis. Mammography eligibility was defined according to the 2016 USPSTF criteria as any woman aged 50–74 [26]. Mammography and LCS utilization were determined from structured completed orders and documented completion (imaging reports, clinical notes) within 4 years before LC diagnosis. A 4‐year window was chosen based on two considerations. First, to capture a broader history of recent screening *engagement*, including those who may not perfectly adhere to annual or biennial guidelines. Second, this window is clinically relevant as it aligns with the natural history of the disease. The median preclinical sojourn time (the screen‐detectable, asymptomatic period) for LC in women is estimated at 1.49 years, and the subsequent average symptomatic period before diagnosis is 1.56 years [27, 28]. Our 4‐year window was thus selected to fully encompass this > 3‐year total detectable disease timeframe. Patients without documented screening or those screened more than 4 years before LC diagnosis were deemed unscreened. Finally, we calculated the time interval from LCS or mammography to LC diagnosis. When patient information at diagnosis was unavailable, we captured the data from the closest available date, preferably before the LC diagnosis, since major health events can lead to changes in critical information such as address and insurance type/status.
- *Geographic Classification*: We matched patient zip codes to the 2020 Census Demographic and Housing Characteristics File and accordingly classified them as urban or rural [29].

### 2.4 Outcomes

Our primary outcomes included determining (1) the overall proportion of women with LC who were eligible for and participated in mammography or LCS prior to diagnosis and (2) quantifying the “market viability” of bridging the two screenings, namely, estimating the “total addressable market”: the percentage of women with LC who were eligible for both screenings and the total serviceable market: the percentage of women with LC who were eligible for both screenings and participated in mammography but not LCS. Our secondary outcome involved examining how various patient factors influenced the likelihood of undergoing either mammography or LCS among women with LC who were eligible for both screenings.

### 2.5 Statistical Methods

We generated descriptive statistics for patient characteristics and screening eligibility, and utilization rates. To assess factors associated with screening uptake among dually eligible women, we used chi‐square tests for two binary outcomes: (1) mammography uptake (yes/no) and (2) LCS uptake (yes/no). Variables examined included race (White vs. non‐White), ethnicity (Hispanic vs. non‐Hispanic), marital status (married vs. others), insurance type (private vs. others), area classification (urban vs. rural), employment status (employed vs. other), and LC stage (stage 1 vs. others). We calculated odds ratios with 95% confidence intervals to assess associations between variables and screening uptake. Statistical significance was defined as two‐sided *p* < 0.05 without adjustment for the number of comparisons. Analysis was performed using SPSS 29.

## 3 Results

### 3.1 Patient Demographics and Disease Characteristics (Table 1)

Of the 400 women initially identified with newly diagnosed LC, 345 (86.2%) met the inclusion criteria (Figure 1). The mean age at LC diagnosis was 64.8 (SD = 11.35). The cohort was predominantly White (87.5%), 53.0% had Medicare, and 53.6% resided in urban areas, as defined by US Census criteria. The cohort consisted of 43.2% individuals who currently smoked and 33.0% who formerly smoked. Most patients were diagnosed with Stage IV LC (41.2%), with a combined 55.4% diagnosed at a late stage (Stage III or IV) (Table 1).

**TABLE 1:** Baseline characteristics of women diagnosed with lung cancer during 2019 and 2020. Lung cancer screening eligibility is based on the 2021 USPSTF criteria.

Demographics	Categories	Cohort	Eligible	Not eligible
Total *N*		345	170	175
Mean age (SD) by years		64.8 (11.35)	64.8 (7.0)	64.8 (14.4)
Race	White	302 (87.54%)	155 (91.18%)	147 (84%)
	Black	28 (8.12%)	10 (5.88%)	18 (10.29%)
	Other	15 (4.35%)	5 (2.94%)	10 (5.71%)
Lung cancer stage at diagnosis	I	115 (33.33%)	55 (32.35%)	60 (34.29%)
	II	34 (9.86%)	20 (11.76%)	14 (8.00%)
	III	49 (14.2%)	27 (15.88%)	22 (12.57%)
	IV	142 (41.16%)	67 (39.41%)	75 (42.86%)
	Unknown	5 (1.45%)	1 (0.56%)	4 (2.29%)
Marital status	Single	51 (14.78%)	27 (15.88%)	24 (13.71%)
	Married	169 (48.99%)	86 (50.59%)	83 (47.43%)
	Divorced	63 (18.26%)	35 (20.58%)	28 (16.00%)
	Widowed	60 (17.39%)	22 (12.94%)	38 (21.71%)
	Unknown	2 (0.58%)	2 (1.18%)	0 (0.00%)
Insurer	Medicaid	40 (11.59%)	18 (10.59%)	22 (12.57%)
	Medicare	187 (54.20%)	98 (57.65%)	89 (50.86%)
	Private	104 (30.14%)	44 (25.88%)	60 (34.29%)
	Uninsured	14 (4.06%)	10 (5.88%)	4 (2.29%)
Employment status	Employed	90 (26.09%)	36 (21.18%)	54 (30.86%)
	Unemployed	83 (24.06%)	47 (50.59%)	36 (20.57%)
	Unknown	8 (2.32%)	5 (2.94%)	3 (1.71%)
	Retired	111 (32.17%)	47 (27.65%)	64 (36.57%)
	Disability	53 (15.36%)	35 (20.59%)	18 (10.29%)
Address classification	Rural	160 (46.38%)	85 (50.00%)	75 (42.86%)
	Urban	185 (53.62%)	85 (50.00%)	100 (57.14%)
Smoking status at diagnosis	Current	149 (43.19%)	118 (69.41%)	31 (17.71%)
	Former	114 (33.04%)	48 (28.24%)	66 (37.71%)
	Never	75 (21.74%)	NA	75 (42.86%)
	Unknown	7 (2.03%)	4 (2.35%)	3 (1.71%)
Simplified pack year at diagnosis	Never smoked	75 (21.74%)	NA	75 (42.86%)
	*X* < 20	38 (11.01%)	NA	38 (21.71%)
	20 < *X* < 30	26 (7.54%)	20 (11.76%)	6 (34.26%)
	30 < *X*	178 (51.59%)	148 (87.06%)	30 (17.14%)
	Unknown	28 (8.12%)	2 (1.18%)	26 (14.86%)
BCS eligibility	Eligible	253 (73.33%)	156 (91.76%)	97 (55.43%)
	Not eligible	92 (26.67%)	14 (8.24%)	78 (44.57%)

Abbreviations: LC, lung cancer; LCS, lung cancer screening.

### 3.2 LCS and Mammography Eligibility and Utilization Among Women With LC (*N* = 345) (Figure 2)

Of all our cohort, 43.5% (150/345) were LCS‐eligible per the 2013 USPSTF LCS eligibility criteria, with only 13.9% (48/345) undergoing LCS before diagnosis. Among LCS‐eligible (2013) women, 32.0% (48/150) received LCS. Under the 2021 criteria, LCS eligibility increased to 49.28% (170/345).

**FIGURE 2:**
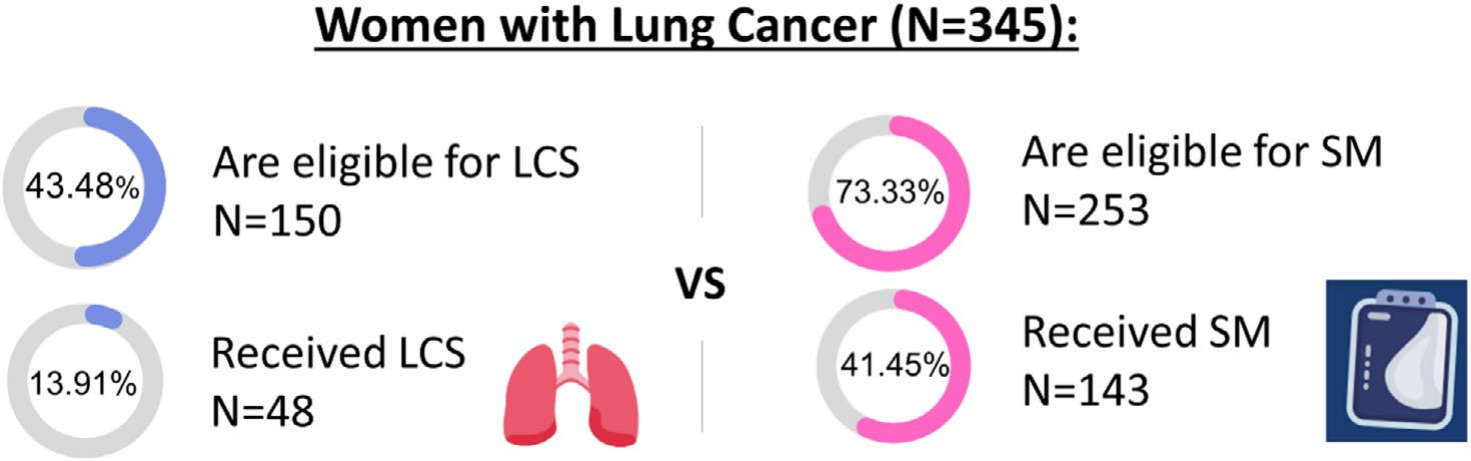
Visual representation of LCS (2013) versus mammography (2016) eligibility and uptake among patients with LC prior to diagnosis. All percentages are based on the same denominator, the 345 eligible women with LC. LC, lung cancer; LCS, lung cancer screening; SM, screening mammography.

For mammography, 73.3% (253/345) of the cohort were eligible, and 41.5% (143/345) underwent mammography in the 4 years preceding their LC diagnosis. The screening rate among mammography‐eligible women was 56.5% (143/253).

Among mammography participants, 48.9% (70/143) were eligible for LCS per the 2021 criteria, representing 20.3% (70/345) of the total cohort and 41.2% (70/170) of all LCS‐eligible cases. The median time from mammography to LC diagnosis was 351.0 days (SD 316.5 days).

### 3.3 The Opportunity Size of Concurrent LCS and Mammography Among Screening Eligible Patients With LC (Figure 3)

**FIGURE 3:**
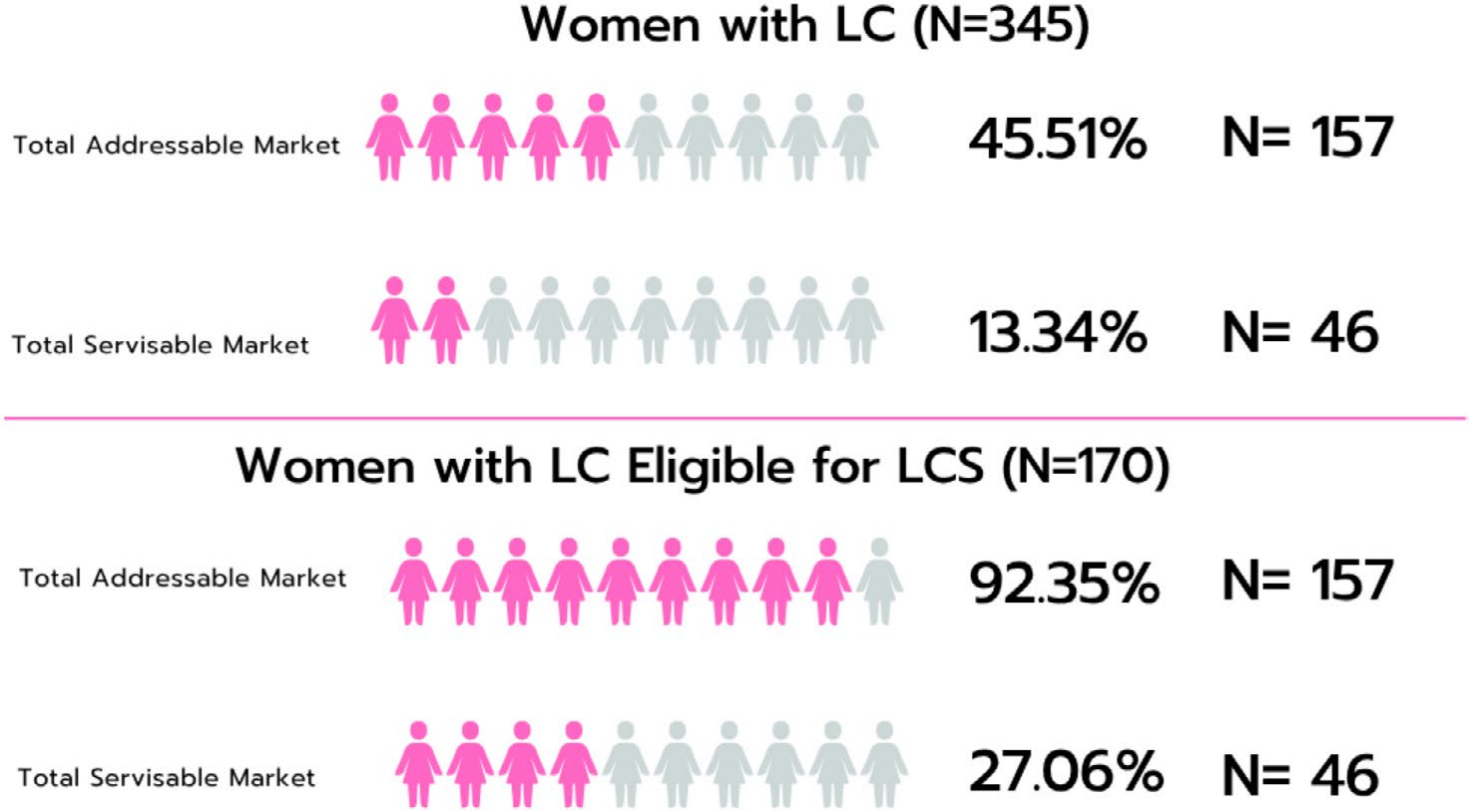
Visual representation of the estimated total addressable market (percentage of women dually eligible for mammography and LCS) and total serviceable market (percentage of women who are eligible for both mammography and LCS, received mammography but not LCS) among women with LC and among LCS‐Eligible women with LC, respectively. LC, lung cancer; LCS, lung cancer screening; SM, screening mammography.

#### 3.3.1 Total Addressable Market

Among all women with LC, 45.5% (157/345) are concurrently eligible for mammography and LCS under the 2021 criteria. This segment represents the maximal LC detection opportunity if all dually eligible individuals received timely combined screening. They constitute 92.4% (157/170) of all LC cases eligible for LCS (2021).

#### 3.3.2 Total Serviceable Market

Among women with LC, 20.3% (70/345) were mammography participants and eligible for LCS, representing the real‐world LC detection opportunity if women with LC who underwent mammography also received LCS before diagnosis. *This group constitutes 41.2% (70/170) of all LCS‐eligible LC cases in our cohort*.

### 3.4 Factors Influencing Screening Uptake Among Dually Eligible (Table 2)

Rural residency was associated with lower LCS uptake (OR, 0.42; 95% CI 0.19–0.94; *p* = 0.031), although it did not affect mammography participation rates (*p* = 0.256). Women who participated in mammography were significantly more likely to undergo LCS (OR, 2.67; 95% CI 1.21–5.87; *p* = 0.013). We observed no significant differences in screening rates for either mammography or LCS across age, race, ethnicity, marital status, insurance, and employment at diagnosis.

**TABLE 2:** The association of demographic and clinical factors on mammography and LCS (2013) uptake among those dually eligible women with LC.

Factor	Category	Mammography	Mammography	LCS	LCS
		Odds ratio and 95% CI	*p*	Odds ratio and 95% CI	*p*
Race	White vs. non‐White	1.333 (0.44–4.07)	*p* = 0.610	2.262 (0.73–6.97)	*p* = 0.162
Ethnicity	Hispanic vs. non‐Hispanic	0.484 (0.21–1.12)	*p* = 0.139	0.784 (0.46–1.35)	*p* = 0.365
Marital status	Married vs. not married	1.166 (0.61–2.23)	*p* = 0.626	0.794 (0.35–1.81)	*p* = 0.556
Insurance type	Private vs. other	1.305 (0.63–2.68)	*p* = 0.469	0.829 (0.35–1.96)	*p* = 0.669
Area classification	Rural vs. urban	1.448 (0.76–2.77)	*p* = 0.256	0.418 (0.19–0.94)	*p* = 0.031*
Employment status at Dx	Employed vs. not employed	1.497 (0.71–3.16)	*p* = 0.123	1.086 (0.44–2.65)	*p* = 0.858
Mammography uptake	Mammography participants vs. mammography nonparticipants	N/A	N/A	2.667 (1.21–5.87)	*p* = 0.013*

*Note:* *Statistically significant (*p* < 0.05).

Abbreviations: BCS, breast cancer screening; CI, confidence interval; Dx, diagnosis; LC, lung cancer; LCS, lung cancer screening.

## 4 Discussion

In this retrospective cohort study of women newly diagnosed with LC, we found several disparities in screening eligibility and utilization patterns. Less than half of women with LC were eligible for LCS, and only a fraction received it prior to diagnosis. By contrast, almost three‐quarters were eligible for mammography, and more than half of those eligible underwent this screening. Most importantly, 27% of all LCS‐eligible women who did not receive LCS participated in mammography before their LC diagnosis, highlighting a sizable but missed opportunity for integrating these screenings to facilitate early LC detection. This integration is further supported by the strong association between receiving mammography and LCS uptake in those dually eligible and by the marked rural–urban disparity in LCS uptake, which was absent for mammography uptake.

Our finding that 43.5% of women with LC were LCS‐eligible per the 2013 USPSTF criteria exceeds the 36.6% rate reported in previous studies [30]. This difference likely reflects a greater smoking prevalence, intensity, and shorter quit durations in our cohort. Both rates highlight a consistent gap in screening guidelines coverage for many women ultimately diagnosed with LC. This coverage gap persisted under the 2021 LCS guidelines with half of the women with LC not meeting eligibility (e.g., never smoked), highlighting a need for more inclusive risk‐based criteria. The 32.0% LCS uptake among eligible women, while much greater than the 4.86% reported in broader studies, indicates significant room for improvement [31]. Several factors may explain this discrepancy in uptake rates: improvement in LCS uptake over time, more comprehensive documentation and capture of LCS uptake in the EHR, or our methodology capturing LCS performed at any point within 4 years before diagnosis. On the patient level, this could be due to greater access to primary care [32]. One study demonstrated that over half of patients with LC lacked an established PCP, which correlated with poor LCS participation [33]. Our findings highlight the potential role of nontraditional care pathways in improving LCS uptake. As many women view their obstetrician/gynecologist as a PCP and as radiologists increasingly engage in population health management and cancer screening, these health professionals may serve as new touch points connecting patients lacking a physician referral or consistent primary care to LCS [34, 35]. This alternative pathway is particularly promising given that over 50% of women in our cohort received mammography a year before LC diagnosis—a period when LC is detectable by screening [27, 36]. This timing represents a critical window for earlier LC detection and intervention. The striking disparity we observed between mammography and LCS participation reinforces previous observations that “Women screened for breast cancer are dying from LC,” illustrating a pressing need and opportunity to promote LCS in this receptive population already engaged in preventative cancer screening [37].

Our “market viability” analysis reveals that mammography programs offer a uniquely efficient pathway to reach women at high risk for LC. Among women with LC who were LCS‐eligible, 92.4% were also mammography‐eligible. Thus, if these screenings were integrated and followed, almost all women with LC who are LCS‐eligible could potentially be identified through mammography programs. Due to this high overlap, every investment to expand mammography's reach could increase LC detection, magnifying the return on investment. More importantly, 27% of LCS‐eligible LC cases occurred in women receiving mammography and who had never received LCS prior to their LC diagnosis. They represent a high‐yield, high‐stakes, readily reachable, and receptive population where strategic intervention could yield substantial improvements in early LC detection. These findings suggest that mammography programs, which already enjoy widespread patient acceptance and robust infrastructure, could serve as an effective gateway to LCS. Ongoing efforts to refine LCS guidelines to expand coverage, such as the American Cancer Society's expanded thresholds, could further increase the proportion of LC cases that could be detected via this dual‐screening approach [38, 39].

We observed that among women eligible for both screenings, mammography participants had significantly higher odds of LCS uptake (OR 2.67, 95% CI 1.21–5.87). This association suggests that LCS‐eligible mammography participants are predisposed to participate in LCS. Such predisposition may stem from higher self‐efficacy and greater access and trust in the medical system [40, 41]. These characteristics make them an ideal target to promote LCS as they may require fewer resources to activate toward additional preventive behaviors compared to those entirely disengaged from the healthcare system.

Rural residence emerged as a significant barrier to LCS uptake (OR 0.42, 95% CI 0.19–0.94), a disparity that did not affect mammography uptake. This discrepancy suggests that while mammography has achieved some equitable geographic penetration over decades of implementation, LCS, a more recent intervention, may be less available in rural areas [42]. This finding aligns with known rural barriers to LCS uptake, such as limited awareness, poor geographic access to LCS programs, and limited provider recommendation [43]. This geographic disparity is also seen when comparing mammography and colorectal cancer screening adherence among women in the US [44]. The relative success of mammography programs in overcoming rural access barriers makes them particularly valuable as potential pathways for LCS promotion in underserved rural communities. By leveraging the existing mammography infrastructure and patient engagement, health systems could potentially address the persistent rural disparities in LCS uptake more efficiently than building standalone LCS outreach programs.

Key strengths of this study include the rigorous examination of demographic and clinical factors influencing screening behaviors using real‐world data from a comprehensive cancer center, which serves some of the counties with the highest smoking prevalence and LC death rates among women nationally [45]. Additionally, our use of EHR‐integrated state Health Information Exchange data allowed for more complete capture of screening history than single‐source records typically permit, yet it is still possible that screenings performed at nonparticipating institutions or out‐of‐state were missed. Limitations of the current analysis include its retrospective nature, single health system, and modest sample size. The predominantly white population also limits the generalizability of our findings, but is representative of other midwestern populations. Furthermore, the study cohort consists of patients already diagnosed with LC at a comprehensive cancer center. These individuals may have had different healthcare access patterns or health‐seeking behaviors compared to the general screening‐eligible population, which could influence the observed screening uptake rates and may not be fully generalizable to all individuals eligible for screening. Despite using the EHR's charts search function to extract the relevant missing smoking information, we could not determine the LCS eligibility status for 7.5% of patients, a small but significant number. This gap showcases the need for better documentation of smoking history information.

Our findings provide key implementation benchmarks for integrating mammography and LCS; even modest success in bridging these screening modalities could substantially reduce LC mortality among women. Prospective implementation studies are warranted to validate these findings and evaluate multimodal approaches tailored to different practice settings. Potential strategies include online tools or telemedicine consultations for LCS eligibility assessment, integrating mobile LCS units into established mammography programs and mobile units, or partnerships between rural primary care and obstetrics and gynecology professionals and urban LCS centers [46]. Looking further ahead, advances in imaging technology may allow radiologists to play a greater role in preventative care through a “one‐stop‐shop” single‐visit multimodal assessment—bundling multiple screenings into a single chest CT to concurrently conduct LCS, coronary calcium scan, chronic obstructive pulmonary disease, bone density and breast density scans [47, 48, 49]. This approach could potentially improve the cost‐effectiveness and patient acceptability of cancer screening programs, particularly for rural women who face significant travel barriers to specialty care.

## 5 Conclusion

Promoting LCS to eligible mammography participants has substantial potential to enhance early LC detection among women. By leveraging the established infrastructure, wide acceptance, and equitable reach of mammography programs, health systems could efficiently identify and engage a significant proportion of women at high risk for LC who are already participating in cancer prevention activities and are receptive to additional screening. The striking rural–urban disparity in LCS uptake—absent in mammography uptake—further emphasizes the strategic value of this approach for reducing geographic inequities in LC outcomes. Future research and implementation efforts should focus on developing, testing, and scaling integrated screening approaches that capitalize on these existing patterns of preventive care engagement to address the persistent challenge of late‐stage LC diagnosis in women.

## Author Contributions

**Ali Ajrouch:** conceptualization (equal), data curation (equal), investigation (equal), methodology (equal), supervision (equal), writing – original draft (equal), writing – review and editing (equal). **Yara Khalifeh:** data curation (equal), investigation (equal). **Amir F. Beirat:** data curation (equal), investigation (equal), writing – original draft (equal). **Dana Alhaffar:** data curation (equal), investigation (equal). **Ahmad Karkash:** data curation (equal), investigation (equal). **Razan Aljaras:** data curation (equal), writing – original draft (equal). **Adel Hajj Ali:** data curation (equal). **Nicholas Pettit:** investigation (equal), methodology (equal), writing – original draft (equal), writing – review and editing (equal). **Deanna R. Willis:** writing – original draft (equal), writing – review and editing (equal). **Victoria L. Champion:** writing – original draft (equal), writing – review and editing (equal). **Lisa Carter‐Bawa:** investigation (equal), writing – original draft (equal), writing – review and editing (equal). **Amrou Awaysheh:** conceptualization (equal), investigation (equal), writing – original draft (equal), writing – review and editing (equal). **Kolawole S. Okuyemi:** funding acquisition (equal), supervision (equal), writing – review and editing (equal).

## Conflicts of Interest

The authors declare no conflicts of interest.

## Data Availability

Raw data were generated at Indiana University. Derived data supporting the findings of this study are available from the corresponding author on request.
